# 12-Oxoeicosatetraenoic acid, a candidate signal for placenta separation, activates matrix metalloproteinase and induces apoptosis in bovine trophoblast cells

**DOI:** 10.5713/ab.22.0097

**Published:** 2022-06-30

**Authors:** Hachiro Kamada

**Affiliations:** 1 Institute of livestock and Grassland Science, NARO, Tsukuba, 305-0901, Japan; 2 Tohoku Agricultural Research Center, NARO, Shimokuriyagawa, Morioka, 020-0198, Japan

**Keywords:** Apoptosis, Bovine, Matrix Metalloproteinase, 12-Oxoeicosatetraenoic Acid, Trophoblast

## Abstract

**Objective:**

12-oxo-5Z,8Z,10E,14Z-eicosatetraenoic acid (12-KETE), a metabolite of arachidonic acid, is a strong candidate signal for placenta separation following calf discharge at delivery. In the present study, the effects of 12-KETE on bovine trophoblast cells were investigated to determine its function in the placentome at delivery.

**Methods:**

Bovine trophoblast cells derived from blastocysts were used. They were cocultured with or without fibroblasts derived from bovine placentome and/or bovine uterine epithelial cells. 12-KETE was added to the culture medium.

**Results:**

Bovine trophoblast cells contained binucleate cells and strongly expressed caudal type homeobox 2 (*CDX-2*) genes. Addition of 12-KETE to the trophoblast cell colony without feeder cells or that on a fibroblast monolayer induced rapid exfoliation of the colony. After 12-KETE addition, trophoblast cells emitted strong fluorescence caused by the degradation of dye-quenched collagen, indicating that 12-KETE activated matrix metalloproteinase of the trophoblast cells. Exfoliated cell colonies were stained with YOPRO-1, but not propidium iodide (PI). Moreover, DNA fragmentation and Bcl-2 associated X protein (*Bax*) gene (apoptosis stimulator) upregulation were observed in exfoliated cells, indicating that 12-KETE induced trophoblast cell apoptosis. These results were consistent with previous *in vivo* observations; however, even a lower concentration of 12-KETE activated trophoblast protease. Meanwhile, fibroblasts derived from the bovine placentome converted arachidonic acid to 12-KETE.

**Conclusion:**

These observations indicate that 12-KETE may serve as a signal for placenta separation at delivery.

## INTRODUCTION

Delivery is completed by placental expulsion after calf delivery. Although the process of calf delivery has been investigated in detail thus far [1], there is little information regarding the separation and expulsion mechanisms of the disused placenta after calf delivery. When cows suffer from retained placenta (RP), they can deliver their calves but cannot discharge their placenta. This means that the mechanism of placental expulsion is different from that of calf delivery. Calf delivery is realized by the uterine contraction functions of prostaglandin (PG) and oxytocin *in vivo*; however, injection of these hormones cannot induce placental expulsion in cows with RP [2,3], and high plasma PG concentrations were reported in RP cows [4]. These reports indicated that the signal for placental expulsion differs from that for calf delivery.

Exfoliation of the placenta at delivery means the histological separation between fetal placenta tissue and maternal part. Previous reports have speculated the involvement of specific matrix metalloproteinases (MMPs) in this separation [5,6]. And the existence of apoptotic cells in the expelled placental tissue [7] and the expression of apoptosis-related genes in the placentome after delivery [8] indicate the possible role of apoptosis in placental expulsion. However, the signal that initiates these functions after fetal discharge at delivery remains unknown.

In a previous study, we successfully induced placental expulsion via the administration of 12-oxo-5Z,8Z,10E,14Z-eicosatetraenoic acid (12-KETE), a metabolite of eicosatetraenoic acid (Ara), to delivery-induced cows [9]. 12-KETE is a strong candidate of signal for placental expulsion at delivery. Our previous paper showed the response of fibroblasts derived from placentome to 12-KETE [9]. In the present study, we investigated the response of trophoblast cells (a model of placenta cell) derived from bovine blastocyst to 12-KETE.

## MATERIALS AND METHODS

### Preparation of trophoblastic cells derived from bovine blastocyst

*In vivo*-derived embryos were flushed from superovulated Japanese Black donor cows. Superovulation was induced using the following method. Cows were inserted with an intravaginal progesterone releasing device (controlled internal drug release: CIDR; Pfizer, Tokyo, Japan) on Day (–9), and treated with 0.8 mg of estradiol benzoate (OVAHORMON INJECTION; ASUKA Animal Health Co. Ltd., Tokyo, Japan) on Day (–8), a total of 20 armor units (A.U.) of follicle-stimulating hormone (ANTORIN-R•10; Kyoritsu Seiyaku Co., Tokyo, Japan) twice daily at decreasing doses (5, 5, 3, 3, 2, and 2 A.U., in that order) on Day (−4 to −2), and 0.75 mg of cloprostenol (RESIPRON-C; ASUKA Animal Health Co. Ltd., Japan) on the morning of Day (–2). The CIDR was removed on the evening of Day (–2). On the morning of Day 0, 100 μg of the gonadotropin-releasing hormone analog, fertirelin acetate (Supolnen; Kyoritsu Seiyaku Co., Japan) was administered (Day 0) and artificial inseminations (AI) were performed on the evening of the same day and on the morning of Day 1. On Day 7, after the first AI, the embryos were recovered by uterine flushing. Embryos with Code 1 according to the criteria of the International Embryo Transfer Society [10] were cultured in 10 μL of droplets (one oocyte in each droplet) of 25 mM Hepes buffered TCM 199 (GIBCO Life Technologies Inc., Grand Island, NY, USA) supplemented with 5% newborn calf serum (Biowest SAS, Nuaillé, France) until hatching.

Dulbecco’s modified eagles medium (DMEM; Sigma-Aldrich, St.Louis, MO, USA) containing 10% fetal bovine serum (FBS) was used for cultivation of blastocysts and feeder cell culture. Uterine epithelial cells (2.5×10^5^) and placental fibroblast cells (2.5×10^5^) were mixed and seeded onto a 6-well dish. After formation of a confluent monolayer, hatched blastocysts were seeded on the mixed culture. Blastocysts formed its cell colony and then produced floating small vesicles, which were used for subculture and subsequent experiments. Subcultured trophoblast cells were cryopreserved using a slow freezing vessel (BICELL; Nihon Freezer, Tokyo, Japan) and cryopreservation medium (CELLBANKER 1; Nippon Zenyaku Kogyo Co. Ltd., Koriyama, Japan).

All of the procedures were approved by the Animal Care and Use Committee of the Institute of Livestock and Grassland Science NARO.

### Preparation of placental fibroblasts and uterine epithelial cells

Fibroblast cells derived from the bovine placentome were prepared by a previously described method (collagenase dispersion) [9]. The placentomes were collected from 9-month pregnant Holstein cows (n = 1) at a slaughterhouse. Cotyledonary villi tissues were aseptically separated from the membrane and minced on a clean bench. The minced tissue was digested with collagenase solution (Yakult Co., Ltd., Tokyo, Japan) in a spinner flask. The dispersed cells in the supernatant of the digesta were collected by centrifugation. Bovine endometrial epithelial cells were kindly provided by Dr. Takahashi (NARO, Tsukuba, Japan) [11].

### Construction of a double layer of placental fibroblasts and trophoblasts

Placental fibroblasts were seeded onto a 6-well dish (Corning, NY, USA) and cultured to form a confluent monolayer. Subsequently added trophoblast vesicles onto these dishes formed its cell colony layered over the fibroblast monolayer within a few days.

### Quantitative polymerase chain reaction

**Figure 1:**
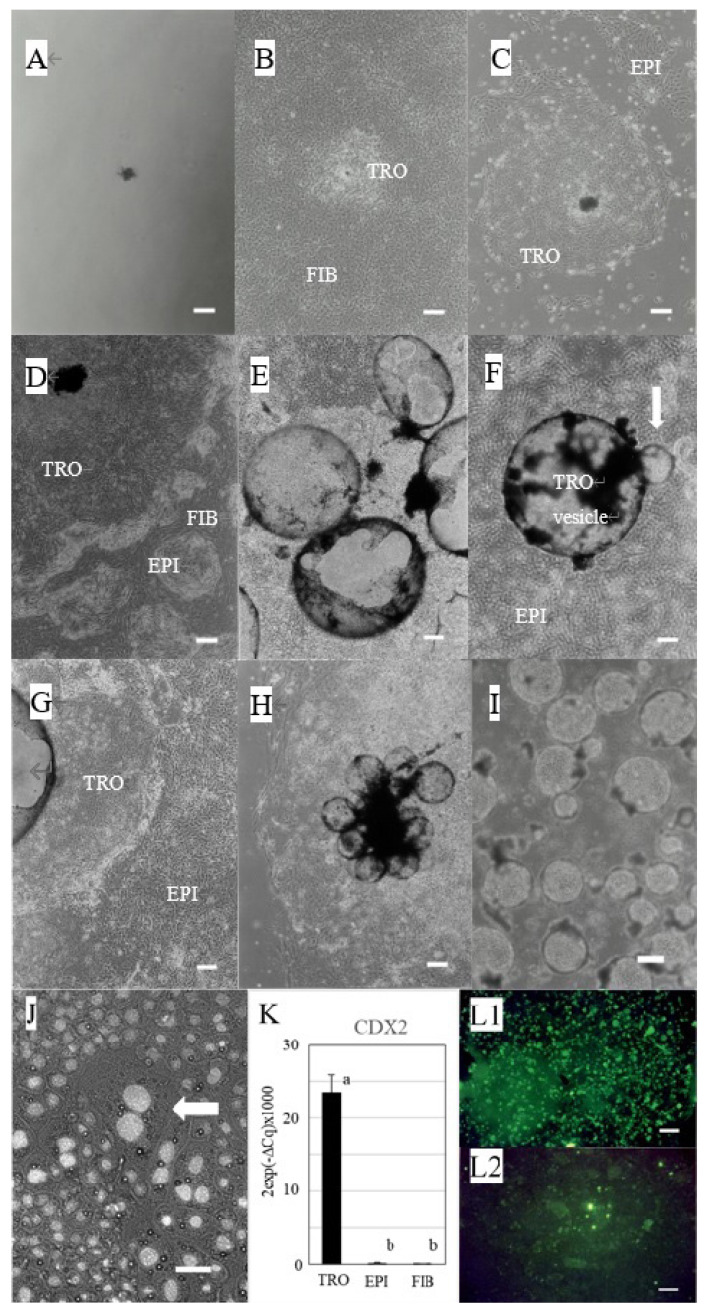
Preparation of trophoblast cells derived from bovine blastocyst. (A) Regressed blastocyst (day 5 of culture), (B) TRO in a culture of FIB derived from the placentome (day 30 of culture), (C) TRO in a culture of uterine epithelial cells (day 5 of culture), (D) TRO in a mixed culture of uterine EPI and FIB derived from the placentome (day 5 of culture), (E) Formation of vesicles (day 25 of culture), (F) Formation of baby vesicles (white arrow) on the surface of trophoblast vesicle in EPI culture (day 13 after transplantation). (G) Formation of the trophoblast colony in EPI culture (day 33 of culture), (H) Trophoblasts colony without feeder cells (day 22 after transplantation), (white bars in A–H: 200 μm). (I) Floating vesicles (white bar: 100 μm). (J) Hoechst staining + phase-contrast image. White arrow shows a binucleate cell, (white bar: 50 μm). (K) qPCR results of *CDX2* gene expression (TRO: n = 5, EPI: n = 3, FIB: n = 3). TRO, trophoblast colony; FIB, fibroblasts; EPI, epithelial cells; CDX2, caudal type homeobox 2. ^a,b^ Different lowercase letters indicate statistically significant differences among cell types (p<0.001). (L1, L2) Immunostaining (white bar: 100 μm). L1, cytokeratin; L2, vimentin.

Total RNA was extracted using the spin column method (PureLink TM RNA Mini Kit; Invitrogen, Carlsbad, CA, USA) 4 h after 12-KETE addition to the culture. RNA concentration was determined using a Qbit fluorometer (Invitrogen, USA). After denaturation at 65°C for 5 min and quenching, the same amount of RNA was used for reverse transcription and template cDNA synthesis using oligo dTs and a random primer mixture (ReverTra Ace qPCR RT Master Mix; TOYOBO, Tokyo, Japan). Quantitative polymerase chain reaction (qPCR) amplification was performed using LightCycler 96 (Roche Diagnostics, Basel, Switzerland). Each 20 μL reaction system contained 2 μL of complementary DNA, 10 μL of PCR MasterMix (FastStart Essential DNA Green Master; Roche Diagnostics, Switzerland), 0.2 μL each of specific reverse and forward primers, and 7.6 μL of RNase free water. PCR was performed at 95°C for 10 min, followed by 45 amplification cycles, each involving denaturation at 95°C for 10 s, annealing at 60°C or 63°C for 10 s, and extension at 72°C for 10 s; and finally an additional melting step at 95°C for 10 s, 65°C for 60 s and 97°C for 1 s. Primer sequences used in this experiment were as follows: glyceraldehyde 3-phosphate dehydrogenase (GAPDH) (forward, 5′-CAGCAATGCATCCTGCAC-3′ and reverse, 5′-GAGTTGCTGTTGAAGTCACAGG-3′) [12], Bcl-2 associated X protein (Bax) (forward, 5′-GCCCCTGTCGTCGTCCTT TGTCC-3′ and reverse, 5′-TGGCGAGGAGCTGGTGCTGG -3′) [12], caudal type homeobox 2 (CDX2) (forward, 5′-GC CACCATGTACGTGAGCTAC- 3′ and reverse, 5′-ACAT GGTATCCGCCGTAGTC-3′) [13], B-cell/CLL lymphoma 2 (Bcl-2) (forward, 5′-CGACTTTGCAGAGATGTCCA-3′ and reverse, 5′-TAGTTCCACAAAGGCATCCC-3′) [12]. The Y-axis in all figures (Figure 1K and 4J) represents the values of 2exp-(ΔCq(target gene-GAPDH)) (Cq: quantification cycles). All melting curves showed a single peak. All primers were purchased from Eurofin Genomics (Tokyo, Japan).

### Fluorescein isothiocyanate-conjugated antibody staining

Prepared cells were used for immunostaining with anti-cytokeratin antibody (Nichirei, Tokyo, Japan) and anti-vimentin antibody (DAKO, Carpinteria, CA, USA). Cultured cells were washed with phosphate-buffered saline (PBS) and then treated with PBS containing 10% formaldehyde (FUJIFILM Wako Pure Chemical Corporation, Osaka, Japan) for 20 min. After washing with PBS containing 1% TritonX-100 (Nacalai tesque, Kyoto, Japan) for 5 min, the cells were treated with a blocking reagent (PBS containing 1% BSA; Sigma-Aldrich, USA) for 30 min at 37°C. The blocked cells were washed three times with PBS containing 0.05% Tween20 (FUJIFILM Wako Pure Chemical Co., Japan) for 10 min. The washed cells were probed with a primary antibody and incubated 4°C overnight. Subsequently, after washing with PBS containing 0.05% Tween20 for 10 min three times, the cells were incubated with secondary antibody (Goat anti-mouse immunoglobulin G [IgG]/fluorescein isothiocyanate [FITC]) (Nordic Immunology, Tilburg, Netherlands) for 30 min at 37°C in the dark. The stained cells were observed under a fluorescence microscopy (IX70; OLYMPUS, Tokyo, Japan).

### Induction of cell exfoliation by 12-KETE

Trophoblast vesicles also formed cell colonies in a culture dish without feeder cells within a few days. Trophoblast cell colony on the fibroblast feeder layer or in the absence of feeder cells were washed with DMEM(−FBS) once and added fresh DMEM(−FBS) containing 4 to 40 μM of 12-KETE (KNC Laboratories Co., Ltd., Kobe, Japan). The culture dish was incubated in 5% CO_2_ and 95% air at 38.5°C. Morphological changes in the colony were observed using a phase contrast microscope (IX70; OLYMPUS, Japan). When the effects of Pefabloc (protease inhibitor) (0.05, 0.2 mg/mL) (Roche, Switzerland) were examined, it was added together with 12-KETE.

### Detection of matrix metalloproteinase activity by dye-quenched collagen

Culture medium of trophoblast cell colony on the fibroblast feeder layer or in the absence of feeder cells was changed to DMEM (+FBS) containing dye-quenched (DQ) collagen (50-fold dilution) (Invitrogen, USA) [14,15]. After incubation for 2 h, the cell colonies were once washed with DMEM (−FBS). And the medium was changed to DMEM (−FBS) containing 4 to 40 μM of 12-KETE. Chromatic images were observed using a fluorescence microscope (IX70; OLYMPUS, Japan).

### Detection of apoptosis by double staining with propidium iodide and YOPRO-1

After 4–5 h of 12-KETE exposure, the cells were incubated with DMEM (−FBS) containing 0.2% YOPRO-1 (Invitrogen, USA) and 0.05% PI (DOJINDO, Kumamoto, Japan) for 20 min. After washing with DMEM (−FBS), chromatic images were observed using a fluorescence microscope (IX70; OLYMPUS, Japan). Cells at an early stage of apoptosis were stained by YOPRO-1 but not by PI [16].

### Detection of DNA fragmentation

*In Situ* Cell Death Detection Kit, Fluorescein (Roche, Switzerland) was used to detect DNA fragmentation. After 24 h of 12-KETE exposure, the culture medium was removed. Air-dried adherent cells in the wells were fixed with 4% paraformaldehyde in PBS (pH7.4) (400 μL) for 60 min at room temperature. After rinsing with PBS, 0.1% Triton X-100 in 0.1% sodium citrate solution (400 μL) was added, and the cells were incubated for 2 min on ice (permeabilization step) and then rinsed twice with PBS. After drying the area around the sample, 200 μL of the the terminal deoxynucleotidyl transferase (TdT)-mediated dUTP nick end-labeling (TUNEL) reaction mixture was added, and the samples were incubated for 90 min at 37°C in the dark. After rinsing with PBS three times, the samples were observed using a fluorescence microscope (IX70; OLYMPUS, Japan).

### Detection of Ara in the uterine epithelial cells

Hydrocortisone at a final concentration of 0.1 and 0.3 μM was added to the culture medium of uterine epithelial cells (5×10^5^/well). After 2 h, the culture medium was discarded, and lysis buffer (10 mM Tris [pH 7.5], 10 μg/mL aprotinin, 10 μg/mL leupeptin, 0.2 mM PMSF, 0.1% sodium dodecyl sulfate) was added to the dish. The lysate was sonicated on ice. Fatty acids in the sample were fluorescently labeled with 9-anthryldiazomethane (Adam; Funakoshi Co. Ltd., Tokyo, Japan) according to following procedure. The same volume of 0.2% Adam/ethyl acetate was added to the sample dissolved in methanol after extraction using the Sep-Pack C18 cartridge column. The mixture was sealed in nitrogen gas and kept at room temperature in the dark overnight. After drying with nitrogen gas, the residue was dissolved in 100 μL of ethyl acetate/methanol (1:1). Reverse-Phase-HPLC (high performance liquid chromatography) (LC-10; Shimadzu, Japan) was performed with a Symmetry C18 column (4.6×250 mm, 5 μm) (Waters, Milford, MA, USA) in acetonitrile/water/phosphoric acid (7:3:0.01) at a flow rate of 1.0 mL/min. The excitation and emission wavelengths were 365 nm and 412 nm, respectively. Organic solvents and other reagents were purchased from FUJIFILM Wako Pure Chemical Corporation (Japan).

### Detection of 12-KETE in the culture medium

Twelve-KETE in the culture medium was measured by HPLC (LC-10; Shimadzu, Japan). The culture medium was collected 90 min after the addition of Ara (FUJIFILM Wako Pure Chemical Co., Japan) at a final concentration of 75 μM to the placenta-derived fibroblast culture. The pH of the medium sample (5 mL) was adjusted to 3. The sample was loaded onto a Sep-Pack C18 cartridge column (Waters, USA), and extracted with 5-mL ethyl acetate/methanol (9:1). After drying with nitrogen gas, the residue was resuspended in methanol. Reverse-Phase HPLC was performed on a Symmetry C18 column (4.6×250 mm, 5 μm) (Waters, USA) in methanol/water/acetic acid (85:15:0.01) at a flow rate of 0.5 mL/min. Absorbance was measured at 280 nm. Organic solvents were purchased from FUJIFILM Wako Pure Chemical Corporation (Japan).

### Statistical analysis

Data were analyzed by analysis of variance using the general linear models procedure (SAS, version 9.2; SAS Institute, Cary, NC, USA). When significant treatment effects were found, Duncan’s multiple range test was used to determine the significance of differences between the treatment groups. The results were considered significant at p<0.05.

## RESULTS

### Culture of trophoblasts

Bovine blastocysts cultured in DMEM containing 10% FBS in the absence of feeder cells regressed within approximately 3 days (Figure 1A). While the use of fibroblasts derived from the bovine placentome as feeder cells enabled the formation of a trophoblast colony; however, this colony was not maintained for a long time (Figure 1B). When uterine epithelial cells were used as feeder cells, blastocysts strongly induced the exfoliation of epithelial cells. Blastocysts rarely attached on the remaining epithelial cells and formed a colony (Figure 1C); however, the colony also was not maintained for a long time. On the other hand, the use of both fibroblast and epithelial cells as feeder cells (mixed culture) enabled the formation of trophoblast colonies within a few days (Figure 1D), and these colonies were maintained for over 6 mo. These colonies actively generated new small vesicles (Figure 1E) that floated and attached at other places in the culture dish and formed new colonies. The floating trophoblast vesicles on uterine epithelial feeder cells, remained floating several days after seeding, and then generated small baby vesicles (white arrow) (Figure 1F). This baby vesicle attached on the epithelial cells and formed a colony (Figure 1G) that grew and produced many floating vesicles; however, epithelial cells under the trophoblast colony disappeared. Therefore, we did not use this combination of cells in subsequent experiments. The floating vesicles could attach to the culture dish in the absence of feeder cells and formed its colony (Figure 1H). These colonies could be subcultured for over 4 yr by transplanting newborn floating vesicle (Figure 1I) or cell colony sheets. Hoechst staining of the subcultured cells showed that these colonies contained binucleate cells, which is a characteristic of trophoblasts (Figure 1J). The results of qPCR showed that the prepared cells highly expressed CDX2 (an essential transcription factor for embryonic development [17]) (Figure 1K). While the expression of CDX2 in uterine epithelial cells and fibroblasts derived from the placenta was very low. And the cells were positively stained with anti-cytokeratin antibody (Figure 1L1), but not anti-vimentin antibody (Figure 1L2).

### Induction of cell exfoliation by 12-KETE

**Figure 2:**
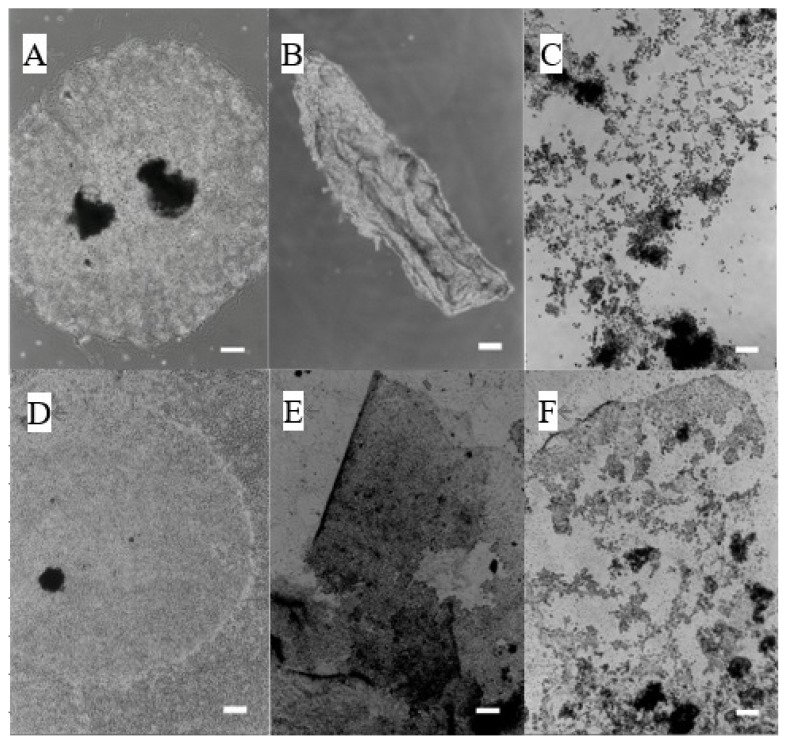
Induction of exfoliation of the trophoblast colony without (A–C) or with (D–F) feeder cells (fibroblasts derived from the placentome) by 12-KETE addition. (A, D) Control, (B) +40 μM of 12-KETE (2.5 h after addition), (E) +40 μM of 12-KETE (5 h after addition), (C) +4 μM of 12-KETE (8 h after addition), (F) +10 μM of 12-KETE (38 h after addition), (white bars: 200 μm). Replications (n≥10) of experiments showed the same results. 12-KETE, 12-oxo-5Z,8Z,10E,14Z-eicosatetraenoic acid.

We used two culture systems (trophoblasts with or without feeder cells) for following experiments. Small vesicles of subcultured trophoblasts formed colonies in the absence of feeder cells in the culture dish (Figure 2A). When 40 μM of 12-KETE, a candidate signal for placenta separation, was added to the culture, the exfoliation of the colony sheet was induced after 2.5 h (Figure 2B). Next, small vesicles of subcultured trophoblasts formed colonies on the fibroblast feeder monolayer (Figure 2D). When 40 μM of 12-KETE was added to the culture, the exfoliation of the colony sheet was induced after 5 h (Figure 2E), and the fibroblast layer under the exfoliated trophoblast colony remained.

### Induction of MMP activation and apoptosis by 12-KETE

**Figure 3:**
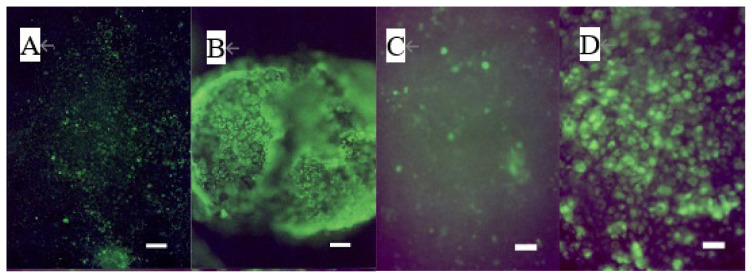
Induction of MMP activation of the trophoblast colony without (n = 3) (A, B) or with (n = 3) (C, D) feeder cells (fibroblasts derived from the placentome) by 12-KETE. Trophoblast colonies were preincubated with dye-quenched collagen for 2 h before 12-KETE addition. (A) No addition of 12-KETE, (B) At 2.5 h after 40 μM of 12-KETE addition, (white bars in A, B: 100 μm), (C) No addition of 12-KETE, (D) At 24 h after 40 μM of 12-KETE addition, (white bars in C, D: 50 μm). Replications (n = 5) of experiments showed the same results. MMP, matrix metalloproteinases; 12-KETE, 12-oxo-5Z,8Z,10E,14Z-eicosatetraenoic acid.

**Figure 4:**
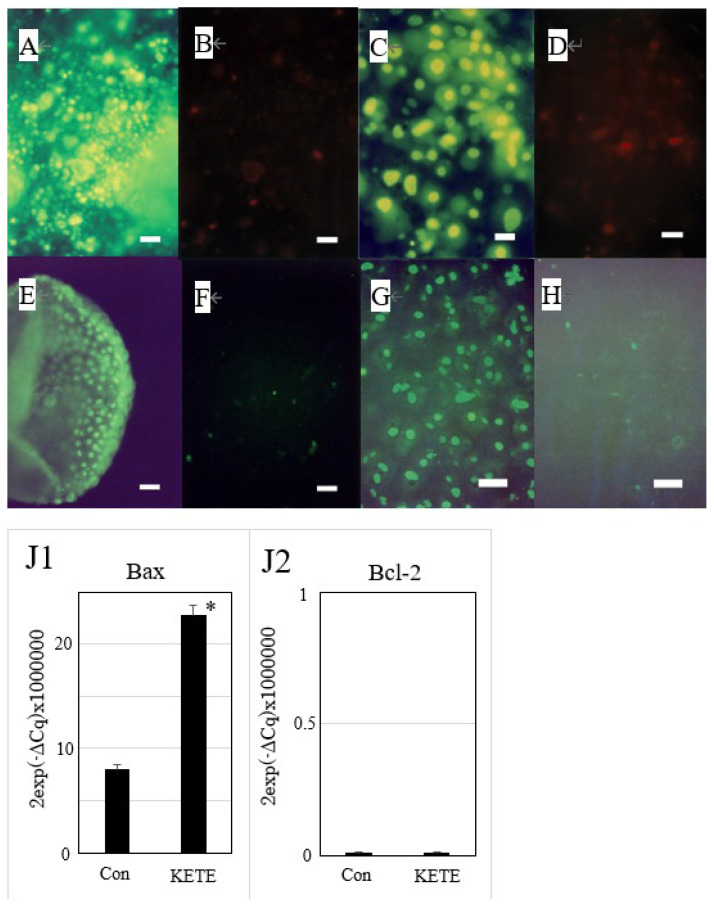
Induction of apoptosis of trophoblast colony in the presence or absence of feeder cells (fibroblasts derived from the placentome) by 12-KETE. Double staining of trophoblast colony without (A, B) (n = 3) or with (C, D) (n = 3) feeder cells by YOPRO-1 (A, C) or PI (B, D), 4 h (A, B) or 5 h (C, D) after 12-KETE treatment (40 μM), (white bars in A, B: 50 μm, in C, D: 25 μm). Detection of DNA fragmentation in trophoblast colony in the absence (E, F) (n = 1) or presence (G, H) (n = 3) of feeder cells. (E) At 19 h after 40 μM of 12-KETE, (F) No addition of 12-KETE, (white bars: 50 μm), (G) At 24 h after 40 μM of 12-KETE, (H) No addition of 12-KETE, (white bars: 50 μm). (J1, J2) qPCR results of *Bax* and *Bcl-2* gene expression. * p<0.05. Replications (n = 3) of experiments showed the same results. 12-KETE, 12-oxo-5Z,8Z,10E,14Z-eicosatetraenoic acid; qPCR, quantitative polymerase chain reaction; *Bax*, Bcl-2 associated X protein; *Bcl-2*, B-cell/CLL lymphoma 2.

The addition of 40 μM of 12-KETE increased the fluorescent intensity of the trophoblast attached DQ collagen beforehand (Figure 3A–D). These observations showed that 12-KETE activated MMPs, thereby separating cell junctions. Next, the role of apoptosis in cell exfoliation was investigated. Trophoblasts were treated with PI and YOPRO-1 5 h after 12-KETE addition. Consequently, trophoblast nuclei treated with 12-KETE were stained with YOPRO-1, but not PI (Figure 4A–D). These observations showed that 12-KETE induced early trophoblast apoptosis. DNA fragmentation, which is an indicator of late apoptosis, was also observed in 12-KETE-treated trophoblasts (Figure 4E–H). qPCR analyses revealed that Bax (apoptosis stimulator) expression was greater in 12-KETE-treated cells than in controls. While Bcl-2 (apoptosis suppressor) expression was extremely low and did not differ between the two treatments (Figure 4J).

### Effects of a low concentration of 12-KETE on trophoblastic colony

**Figure 5:**
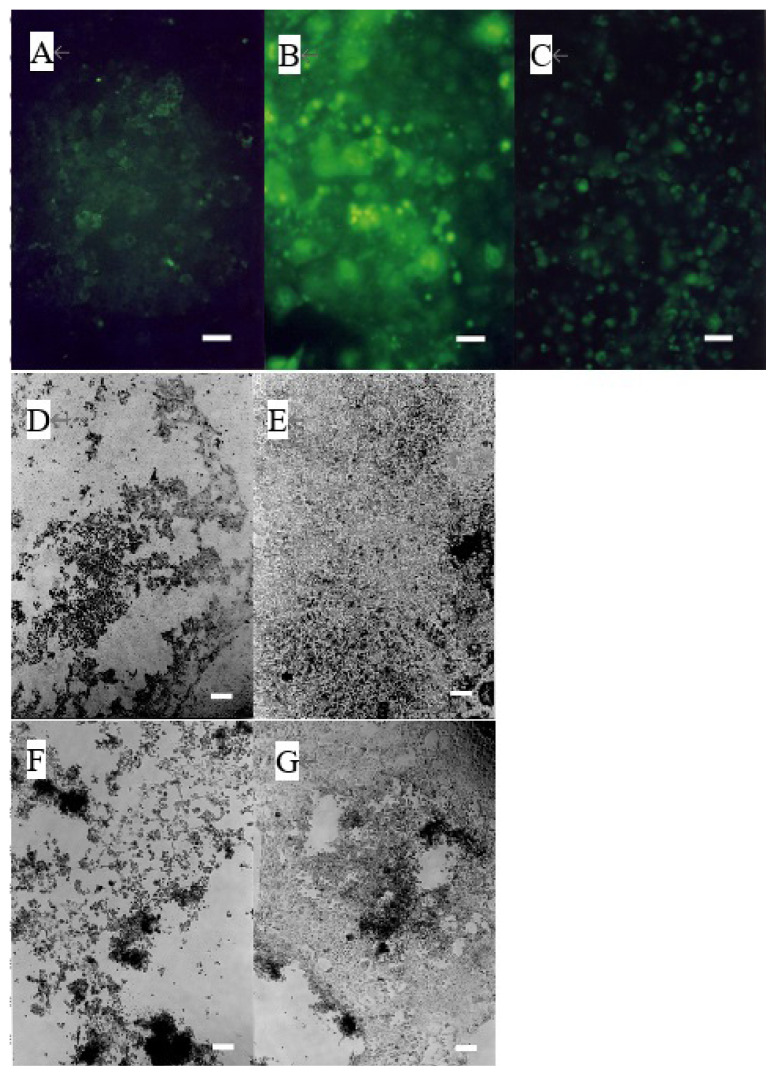
Protease inhibitor-induced suppression of cell exfoliation at a low concentration of 12-KETE. (A) DNA fragmentation (24 h after addition of 10 μM 12-KETE) in the presence of feeder cells (n = 3), (B) YOPRO-1 staining (3 h after addition of 10 μM 12-KETE) in the presence of feeder cells (n = 3), (C) MMP activation (18 h after addition of 10 μM 12-KETE) in the presence of feeder cells (n = 3), (white bars in A, B, C: 25 μm). Results in the absence of feeder cells were the same as those in their presence (data not shown). (D) At 23.5 h after addition of 10 μM 12-KETE in the presence of feeder cells, (E) At 23.5 h after addition of 10 μM 12-KETE plus 0.05 mg/mL Pefabloc in the presence of feeder cells. Replications (n = 3) of experiments showed the same results. (F) At 8 h after addition of 10 μM 12-KETE in the absence of feeder cells, (G) At 8 h after addition of 10 μM 12-KETE plus 0.2 mg/mL Pefabloc in the absence of feeder cells, (white bars in D-G: 200 μm). Replications (n = 4) of experiments showed the same results. 12-KETE, 12-oxo-5Z,8Z,10E,14Z-eicosatetraenoic acid; MMP, matrix metalloproteinases.

The addition of a low concentration of 12-KETE induced a different type of cell exfoliation in the trophoblastic colony. Addition of 10 μM of 12-KETE to the trophoblast colonies layered on fibroblast feeder cells, or 4 μM of 12-KETE to the trophoblast colonies in the absence of feeder cells in culture medium resulted in spheronization and subsequent floating of the trophoblast cells (Figure 2C, 2F). Then MMP activity was low (Figure 5C), and apoptotic cells were rarely observed (Figure 5A, DNA fragmentation; Figure 5B, YOPRO-1 staining). Results in the absence of feeder cells were the same as those in their presence (data not shown). This reaction was suppressed by a protease inhibitor (Pefabloc; Sigma-Aldrich, UK) (Figure 5D–G).

### Detection of free arachidonic acid in uterine epithelial cells

**Figure 6:**
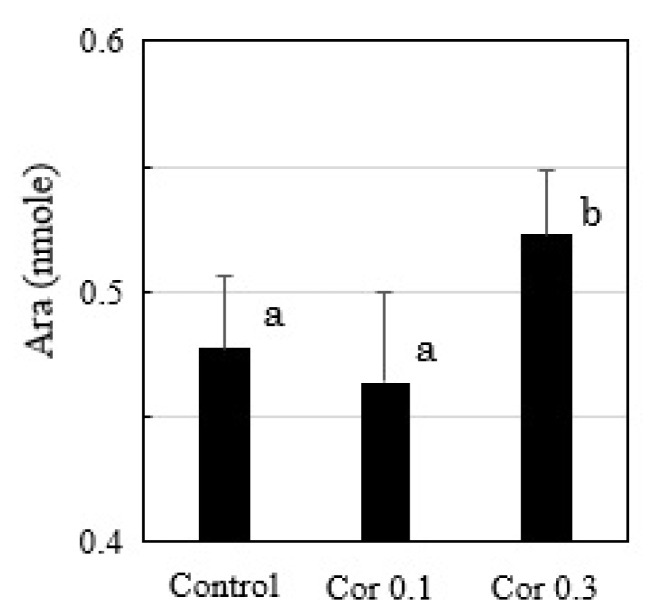
HPLC analysis of free Ara in the cultured uterine epithelial cells with or without hydrocortisone (0.1, 0.3 μM) treatments (n = 3). ^a,b^ Different lowercase letters indicate statistically significant differences among treatments (p<0.05). HPLC, high performance liquid chromatography.

Free Ara was detected in cultured uterine epithelial cells, and its level increased when 0.3 μM of hydrocortisone was added (Figure 6).

### Conversion of arachidonic acid to 12-KETE in fibroblast cells

**Figure 7:**
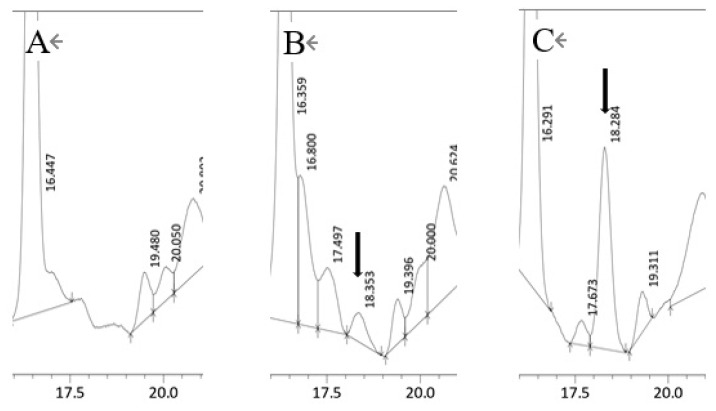
HPLC chromatograph of the fibroblast culture medium. (A) Cultured medium without Ara, (B) Cultured medium containing 75 μM of Ara. (C) Cultured medium spiked with a 12-KETE standard (1.53 ng). Black arrows show 12-KETE peaks. Replications (n = 3) of experiments showed the same results. HPLC, high performance liquid chromatography; Ara, eicosatetraenoic acid; 12-KETE, 12-oxo-5Z,8Z,10E,14Z-eicosatetraenoic acid.

When Ara was added to the fibroblast culture, 12-KETE was detected in the culture medium (Figure 7), suggesting that fibroblast can convert Ara to 12-KETE; however, trophoblast and uterine epithelial cells did not show any conversion activity (data not shown).

## DISCUSSION

In a previous study, we have demonstrated that 12-KETE, a metabolite of arachidonic acid, is a candidate of signal for placenta separation at calf delivery; however, it is difficult to examine the precise mechanism of placenta separation at delivery in large animals. Therefore, we investigated the responses of trophoblasts derived from a bovine blastocyst to 12-KETE as a model of placenta parenchymal cells.

Previous studies on placental expulsion in normal deliveries and RP in cows suggested that MMPs, which are collagen-degrading enzymes *in vivo*, are involved in the separation of bovine placenta at delivery. Maj et al [5] reported a lack of activity of the 64-kDa and 60-kDa active forms of MMP-2 in the maternal and fetal parts of the RP. According to Eiler et al [18] the placentome structure may be supported by collagenous folds of cotyledon engulfing the caruncle stalk, like a purse string.

The present study showed that 12-KETE induced a separation between trophoblasts and fibroblasts derived from the placentome, and a detachment of trophoblasts from the culture dish (Figure 2). This process was mediated via collagen degradation indicating that 12-KETE activated MMPs (Figure 3). These observations supported the hypothesis that 12-KETE is an *in vivo* signal for inducing placenta separation after fetal discharge at delivery through the degradation of intercellular adhesion and collagenous folds. Meanwhile, apoptosis is the well-known *in vivo* mechanism underlying active removal of unused tissues or cells. Twelve-KETE also induced trophoblast apoptosis (Figure 4). Chromatic figures of apoptotic cells were reported in placentas expelled via natural delivery [7]. Therefore, 12-KETE-induced trophoblast apoptosis observed in this *in vitro* study is consistent with the *in vivo* phenomenon.

Previous reports [5,7] suggested the involvement of MMP activity and apoptosis in placental separation process at bovine delivery, however, the present study demonstrated that a lower concentration of 12-KETE activated trophoblast proteases (Figure 5). Then MMP activity was low, and there were few apoptotic cells. These results suggested the involvement of a specific protease in the process of placenta separation at delivery, which is different from the conventional model.

Increased PG production at delivery [19,20] indicates the existence of Ara, which is the precursor of PG, in the uterus [21]. We also confirmed the existence of an Ara pool enable to change by delivery-relating hormone (cortisol) in uterine epithelial cells used in this experiment (Figure 6). Since 12-KETE is synthesized from Ara, it is possible that 12-KETE is produced in the placentome *in vivo*. The present study also showed that fibroblasts derived from the placentome can convert Ara to 12-KETE (Figure 7). A signal for placenta separation at delivery may be supplied by placental fibroblasts. If the metabolic pathway of Ara partly shifts from PG synthesis to 12-KETE synthesis after fetal discharge at delivery, we can understand the mechanism through which separation between the fetal membrane and maternal part occurs timely after fetus discharge. We have detected a 12-KETE peak in the blood plasma of the cows prior to placental expulsion *in vivo* [9]; however, the biochemical trigger that shifts the metabolism of Ara remains unclear. While it is known that 12-KETE is also produced by leukocyte [22,23] and that the chemotactic activity of the placentome in cows with successful placental expulsion was significantly greater than that in the cows with a RP [24–26]. Therefore, there is another possibility that 12-KETE may be supplied by leukocytes at delivery.

The classic signal transduction pathway at delivery is as follows [27–29]: Cortisol is released from the fetus, which triggers delivery. Subsequently arachidonic acid is released by the function of phospholipase and converted to PG. The synthesized PG induces luteolysis and uterine contraction, resulting in calf discharge. However, these previous understanding of signal transduction at delivery lacked the idea for mechanism of placenta discharge. Our hypothesis is as follows. After arachidonic acid release, the delivery signal ramifies into PG synthesis and 12-KETE synthesis which induces MMP activation and apoptosis thereby separating the fetal membrane from maternal tissue. Placental expulsion from the uterus is achieved through this mechanism and uterine contraction. We have already reported the appearance of a 12-KETE peak in the blood plasma of cows prior to placental expulsion [9]. The process of delivery does not end with a calf discharge and is completed by 12-KETE release and subsequent placenta discharge. The present study showed that 12-KETE induced the cleavage of intercellular adhesions in the trophoblast via MMP activation and apoptosis. Our findings could be available for the development of a new method to prevent RP of cows. To progress with this development, further experiments using cows including Holstein and Japanese Black will be needed.
